# Model-Informed Dose Optimization of Spironolactone in Neonates and Infants

**DOI:** 10.3390/ph18030355

**Published:** 2025-03-01

**Authors:** Amira Soliman, Leandro F. Pippa, Jana Lass, Stephanie Leroux, Valvanera Vozmediano, Natalia V. de Moraes

**Affiliations:** 1 Center for Pharmacometrics and Systems Pharmacology, Department of Pharmaceutics, University of Florida, Orlando, FL 32827, USA; amira.soliman@ufl.edu (A.S.); pippalf@gmail.com (L.F.P.); 2 Department of Pharmacy Practice, Faculty of Pharmacy, Helwan University, Helwan 11795, Egypt; 3 Institute of Pharmacy, University of Tartu, 50411 Tartu, Estonia; jana.lass@kliinikum.ee; 4 Pharmacy Department, Tartu University Hospital, 50406 Tartu, Estonia; 5 CHU Rennes, University Hospital Rennes, Inserm, EHESP, Irset—UMR_S 1085, F-35000 Rennes, France; stephanie.leroux013@gmail.com; 6 Model Informed Development, CTI Laboratories, Covington, KY 41011, USA

**Keywords:** PBPK, infants, pediatrics, spironolactone, dose optimization

## Abstract

**Background/Objectives**: Spironolactone (SP) has been used off-label in pediatrics since its approval, but its use is challenged by limited pharmacokinetic (PK) data in adults and especially in children. **Methods**: Physiologically based pharmacokinetic (PBPK) models for SP and its active metabolites, canrenone (CAN) and 7α thio-methyl spironolactone (TMS), in adults were developed. These models aim to enhance understanding of SP’s PK and provide a basis for predicting PK and optimizing SP dosing in infants and neonates. Given SP’s complex metabolism, we assumed complete conversion to CAN and TMS by CES1 enzymes, fitting CES1-mediated metabolism to the parent-metabolite model using PK data. We incorporated ontogeny for CES1 and CYP3A4 and other age-related physiological changes into the model to anticipate PK in the pediatric population. **Results**: The PBPK models for SP, CAN, and TMS accurately captured the observed PK data in healthy adults across various dosing regimens, including the impact of food on drug exposure. The pediatric PBPK model was evaluated using PK data from infants and neonates. Simulations indicate that 2.5 mg/kg in 6-month to 2-year infants and 2 mg/kg in 1–6-months infants matched the total unbound systemic exposure equivalent to the standard recommended daily maintenance dose of 100 mg in adults for treating edema. **Conclusions**: The developed PBPK model provides valuable insights for dosing decisions and optimizing therapeutic outcomes, especially in populations where clinical studies are challenging.

## 1. Introduction

Spironolactone (SP) is a potassium-sparing diuretic that competes for cytoplasmic mineralocorticoid receptors in the distal tubules of the kidneys. It promotes the excretion of sodium and water while retaining potassium [1]. Oral SP is prescribed for adults to treat various edematous conditions, including heart failure, cirrhosis, nephrotic syndrome, essential hypertension, and primary hyperaldosteronism [2]. Additionally, it has become a standard adjunct in diuretic regimens for children with heart failure and for infants with chronic lung disease, helping to alleviate pulmonary congestion [3]. Despite its widespread use in pediatrics, SP has been used off-label in this population since its approval. Many studies have utilized pharmaceutical compounding of liquid oral dosage forms or powders for extemporaneous use to facilitate administration in infants and children [4,5]; however, no dedicated commercial formulation is currently available for this specific population. Consequently, commercially available formulations are often altered to be administered in children as solutions or suspensions, which can lead to dosing errors, inaccurate dosing, and issues with drug stability and bioavailability. The major adverse effects of spironolactone were alterations in potassium. Therefore, potassium concentrations should be carefully monitored, particularly in children receiving multiple diuretics [3].

SP is a synthetic steroid with complex pharmacokinetics (PK). Due to its poor water solubility (0.022 mg/mL), it is only available in oral formulations. When taken orally, SP is partially absorbed, but micronized formulations have improved absorption, achieving an oral bioavailability of 60–90% [6]. Furthermore, concurrent food consumption enhances bioavailability by promoting absorption and reducing the first-pass effect [7,8]. As a pharmacologically active prodrug with a short plasma half-life of less than two hours, SP undergoes rapid and extensive metabolism in the liver, resulting in at least 17 metabolites. The primary active metabolites are 7α-thiomethylspironolactone (TMS) and canrenone (CAN) [7,9,10]. The thioacetate group in SP undergoes hydrolysis by carboxylesterases, producing 7α-thiospirolactone as an intermediate. This compound is then S-methylated to form TMS by thiol methyltransferase, which is also expressed in human red blood cell (RBC) membranes. Additionally, 7α-thiospirolactone can be converted to CAN, a non-sulfur metabolite of SP. TMS undergoes hydroxylation by CYP3A4 to form 6-OH-7α-thiomethylspironolactone, another active metabolite, and can also be oxidized to 7 α-methylsulfinyl- and 7 α-methylsulfonylspirolactone [7,11]. CAN undergoes further metabolism through three main pathways: hydrolysis of its γ-lactone ring to form canrenoate, which exists in an enzymatic equilibrium with CAN; hydroxylation to 15α-OH-canrenone; and reduction to various di-, tetra-, and hexa-hydro derivatives [7,11]. SP binds strongly and almost equally to human serum albumin (HAS) and α-1 acid glycoprotein (AGP), while CAN binds strongly only to HAS [12]. No experimental data on protein binding are available for TMS.

Despite being available for over 60 years, comprehensive research on SP’s clinical PK, efficacy, and safety remains limited, leading to persisting knowledge gaps. Characterizing the physiological differences in the PK of SP between children and adults is essential for optimizing pediatric dosing. Recognizing this need, the Best Pharmaceuticals for Children Act (BPCA) has identified SP as a priority for pediatric therapeutic trials aiming to assess safety and efficacy [13], and the Food and Drug Administration (FDA) has included SP on its list of drugs requiring pediatric studies [3]. To date, only a recent PK study investigated the PK of SP and its active metabolites in newborns up to two years of age [14]. Model-informed dose optimization in pediatrics integrates patient-specific and drug-specific data to tailor dosing. Physiologically-based pharmacokinetic (PBPK) modeling in pediatrics has proven helpful in optimizing clinical study designs, predicting initial doses in children, and informing dosage decisions [15,16]. Therefore, the objectives of this study are: (1) to establish and evaluate a whole-body parent-metabolite PBPK model of SP and its active metabolites CAN and TMS in adults to enhance our understanding of SP’s complex disposition; (2) to scale the adult PBPK model to pediatrics for the assessing plasma concentration–time profiles; and (3) to use the developed pediatric PBPK model to optimize dosing strategies for neonates and term neonates.

## 2. Results

**Figure 1:**
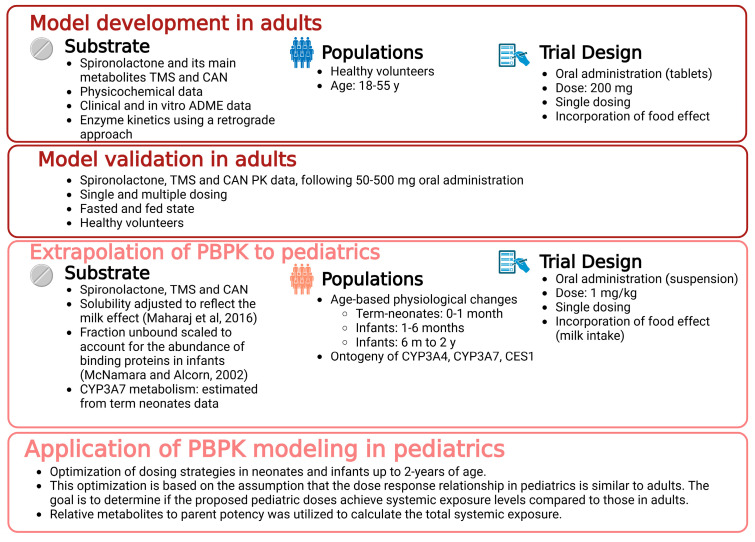
A systematic PBPK modeling workflow was used to predict exposure to spironolactone and its active metabolites in pediatric patients. The model was pragmatically developed and validated in adults before being scaled to the pediatric population. This approach supports dosing recommendations of oral spironolactone for term neonates up to infants aged 2 years. TMS: 7α-thiomethylspironolactone; CAN: canrenone.

The PBPK modeling workflow that was followed to predict exposure to spironolactone and its active metabolites in pediatric patients is presented in Figure 1.

### 2.1. Adult PBPK Model Development and Validation

The parent-metabolite model was developed based on clinical PK data for SP and its main active metabolites, CAN and TMS, following oral administration of SP [17]. A comprehensive literature search identified ten PK studies in adults, which involved single-dose administrations of SP ranging from 50 mg to 200 mg. Additionally, two studies reported multiple-dose administrations of 100 mg of SP. Of these studies, only three provided PK profiles for both the parent compound (SP) and the active metabolites CAN and TMS. Table 1 summarizes the clinical PK studies used for PBPK model development and verification, detailing study characteristics and dosing regimens in adults.

**Figure 2:**
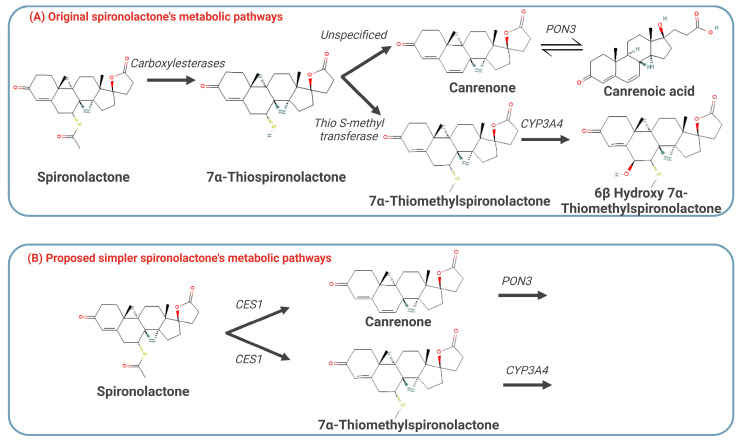
Schematic diagram showing the initial steps of the two main pathways of biotransformation of spironolactone in the human liver (**A**) and the simplified metabolic pathway proposed for modeling purposes (**B**). Only the compounds included in the PBPK model were added to panel B. Panel A is adapted from Varin et al., 1992 [26]. CES1: carboxylesterases1; PON3: Paroxonase3.

**Figure 3:**
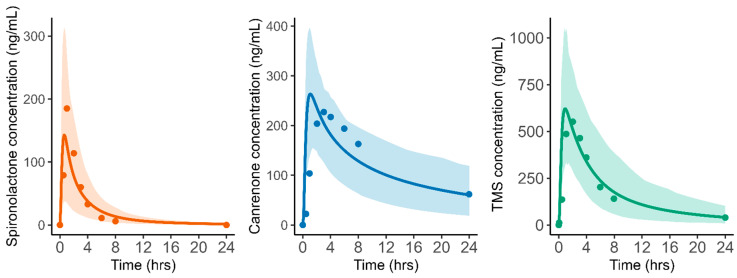
PBPK model development; predicted versus observed plasma concentrations versus time obtained after oral administration of 200 mg of spironolactone tablets for spironolactone (red), canrenone (blue), and TMS (green). Observed data; Overdiek et al. study [17]. The solid lines represent the population means, and the shaded area is the 90% population prediction interval. Observed data are shown as circles representing the mean values.

The drug-dependent parameters of the final PBPK model for SP, CAN, and TMS are presented in Table 2. Detailed information on spironolactone absorption parameters is presented in Table S1. The bile–micelle partition coefficients (log K_m:w_) for the unionized species of SP were estimated in Simcyp™ SIVA to be 4.046. Using the estimated log K_m:w_, the predicted solubility of SP in FaSSIF and FeSSIF media closely matches the experimental solubility values (Figure S1 in Supplementary Materials). Particle size was identified as a critical determinant of dissolution rates, with smaller particles significantly enhancing SP’s dissolution and subsequent absorption. Since no significant differences in exposure were observed between Aldactone^®^ and a micronized powder of 4.29 [25], a monodispersed particle size of 4.29 µm was used for the adult PBPK model. Due to uncertainties in TMS’s fraction unbound in plasma (f_u_) and logP, along with the high sensitivity of the predicted Vss to these parameters, a Kp scalar of 0.51 was fitted to recover a Vss of 2.178 L/kg. Figure 2 illustrates the original metabolic pathway of SP and the proposed assumption used to simplify the elimination model. To build the model, ten virtual trials of 10 subjects (age 18–45 years, male only) receiving a single oral dose of 200 mg of SP were generated, and the predicted and observed PK parameters of SP, CAN, and TMS [17] were compared. The whole-body PBPK model successfully recapitulated the shape and magnitude of the observed profiles for SP, CAN, and TMS following the administration of 200 mg of SP in the fed state [17], as demonstrated in Figure 3.

**Table 2:** Drug-dependent parameters of the final parent–metabolites physiologically based pharmacokinetic (PBPK) model in Simcyp.

Parameter	Spironolactone (SP) Model		Canrenone (CAN) Model		7α-thiomethyl Spironolactone (TMS) Model		
	Value	Source	Value	Source	Value		Source
MW (g/mol)	416.6	[27]	340.5	[28]	388.6		[29]
Compound type	Neutral		Neutral		Neutral		
log*P*	2.78	[27,30]	2.68	[28]	3.2		[29]
*B/P*	0.58	[31]	0.54	[31]	0.58		Assumed the same as SP
*f*_u_ adult	0.14556	Predicted	0.1588	Predicted	0.09943		Predicted
*f*_u_ infants	0.201	McNamara and Alcorn method [32]	0.218	McNamara and Alcorn method [32]	0.140		McNamara and Alcorn method [32]
*f*_u_ neonates	0.226	McNamara and Alcorn method [32].	0.245	McNamara and Alcorn method [32]	0.159		McNamara and Alcorn method [32]
**Absorption**							
Absorption model	ADAM model		ADAM model		ADAM model		
*f*_u, gut_	1	Default value in Simcyp	1	Default value in Simcyp	1		Default value in Simcyp
**Distribution Parameters**							
Distribution Model	Full PBPK		Full PBPK		Full PBPK		
*V_ss_* (L/Kg)	2.81	Simcyp predicted	2.53	Simcyp predicted	2.1779		fitted
*K_p_* Prediction	Method 1 (Poulin and Theil)		Method 1 (Poulin and Theil)		Method 1 (Poulin and Theil)		
*K_p_* scalar	1	Default	1	Default	0.51		fitted
**Elimination Parameters**							
Elimination model	Enzyme kinetics		Enzyme kinetics		Enzyme kinetics		
Metabolism	Esterase mediated pathways (Microsomal) CES1 CLint CAN metabolite = 280 μL/min/mg protein TMS metabolite = 800 μL/min/mg protein	Fitted to observed PO data from Overdiek et al. [17]	PON3 User-esterase (Microsomal) CLint = 17.111 μL/min/mg protein	Simcyp Predicted ^$^	CYP3A4-mediated pathway (Recombinant) = 0.69084 μL/min/pmol		Simcyp Predicted ^$$^
					CYP3A7 mediated pathway (Recombinant) = 1.14 μL/min/pmol		Fitted from term neonates data, Lass et al. [14]
Ontogeny equation	CES1	Simcyp implemented	PON3	No ontogeny	CYP3A4 (profile 1)	CYP3A7	Simcyp implemented
F_birth_	0.205		1		1.06	0	
Adult_max_	1		1		0.11	33	
Age_50_	0.542		10		0.64	0.141	
n	0.977		1		1.91	2.76	
Age cap	25		0		25	25	

MW: Molecular weight, B/P: Blood-to-plasma ratio, f_u_: Fraction unbound (plasma), f_u,gut_: Fraction unbound in the enterocytes, V_ss_: Volume of distribution at steady state, K_p:_ Tissue–plasma partition coefficient, F_birth_: fraction of adult enzyme-specific abundance present at birth, Adult_max_: maximal fraction of adult abundance, Age_50_: age at which half of Adult_max_ is achieved, n: slope factor. ^$^ Simcyp predicted using the reverse translational tool (RTT) based on vivo oral clearance = 9.7 L/h (CAN profile from Overdiek et al. [17]). ^$$^ Simcyp predicted using the reverse translational tool (RTT) based on in vivo oral clearance = 42.3 L/h (TMS profile from Overdiek et al. [17], and accounting for 100% CYP3A4 contribution of hepatic clearance). Ontogeny function: F = $(\frac{{Adult}_{max}-F_{birth}}{{{Age}_{50}}^{n}+{Age}^{n}})\times {Age}^{n}+F_{birth}$.

**Figure 4:**
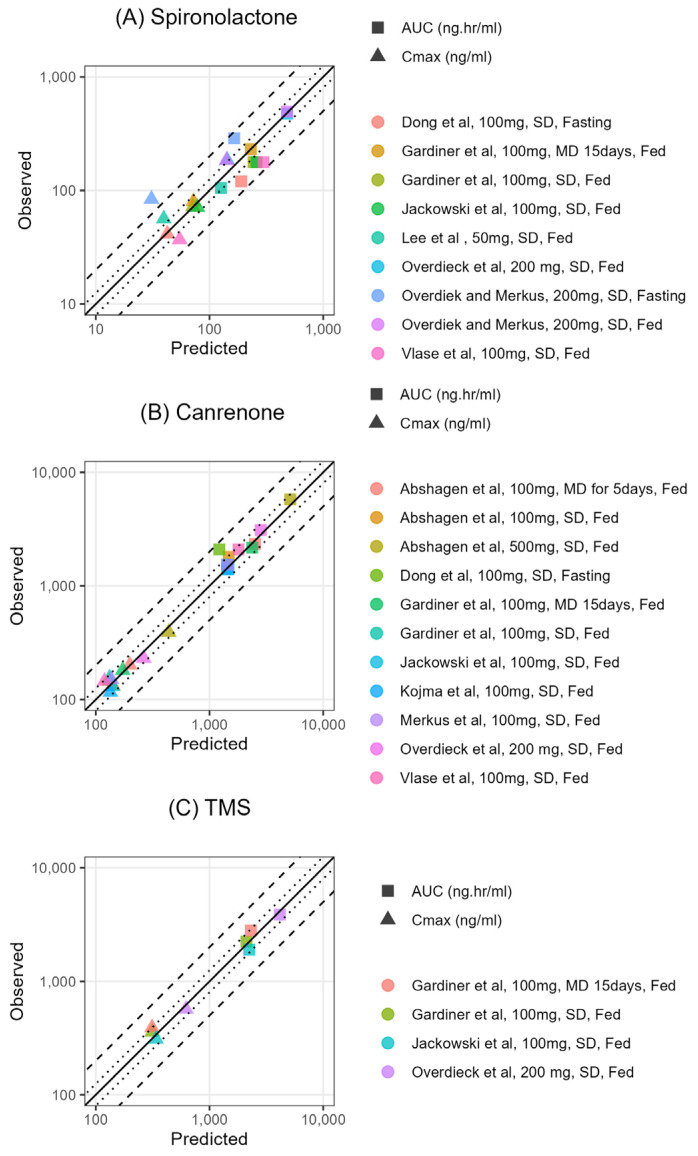
Goodness-of-fit (GoF) plots of AUC_0–last_ and C_max_ predicted versus observed pharmacokinetic metrics for spironolactone (**A**), canrenone (**B**), and 7α-thiomethyl spironolactone (**C**) in adults [7,8,10,18,19,20,21,22,23,24]. The line of identity is shown as a solid line; the 1.25-fold deviation is shown as a dotted line; the 2-fold deviation is shown as a dashed line. AUC_last_: area under the plasma concentration–time curve from the first to the last data point, C_max_: maximum plasma concentration.

The PBPK model for adults was then validated using clinical PK data (Table 1), and the plasma concentration–time profiles and PK parameters of SP, CAN, and TMS were accurately predicted (Figure 4, Supplementary Materials Table S2 and Figure S2). The predicted-to-observed ratios for C_max_ and AUC_last_ for SP, CAN, and TMS fell within the acceptable two-fold range. However, the C_max_ for SP in the fasting state reported by Overdiek and Markus [8] was an outlier at 0.37, which may be attributed to variable formulation bioavailability and/or food effects. The predicted-to-observed ratios for C_max_ and AUC_last_ for CAN and TMS were also within the acceptable range of 0.5–2. The GoF plots illustrate the alignment between the predicted versus observed PK metrics, particularly for AUC_last_ and C_max_ (Figure 4).

### 2.2. Pediatric PBPK Model Development, Validation, and Application

**Figure 5:**
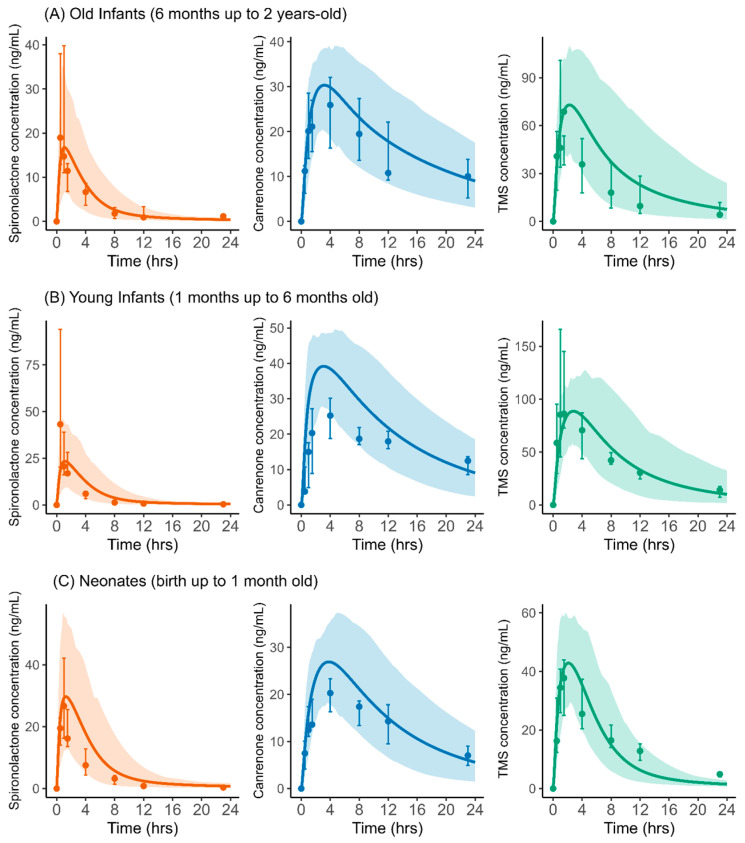
PBPK Model validation in pediatrics. Predicted versus observed plasma concentrations over time obtained after oral administration of 1 mg/kg of spironolactone suspension for spironolactone (red), canrenone (blue), and TMS (green). Observed data; Lass et al. [14]. The solid lines represent the population median, and the shaded areas represent the 90% population prediction interval. Observed data are shown as circles representing the median with error bars representing the 25–75th percentiles range.

**Figure 6:**
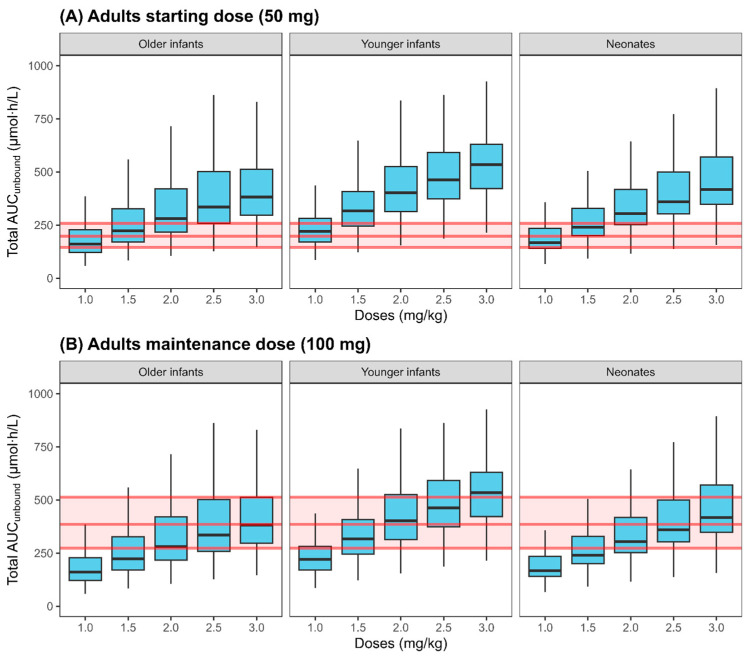
Simulations in infants (older infants: 6 months to 2 years old) (younger infants: 1 month to 6 months old) and neonates (birth to 1 month old), on day 7th following spironolactone 1–3 mg/kg doses (in 0.5 mg increments), administered once daily as an oral suspension, compared to adult exposure to (**A**) starting dose of 50 mg and (**B**) maintenance dose of 100 mg. The total exposure is defined as the unbound plasma AUC of SP, CAN, and TMS normalized by relative potencies of the active metabolites relative to the parent compound.

The validated adult PBPK model for SP, CAN, and TMS was scaled to predict exposure in infants and neonates. Table 3 summarizes the infants’ demographics from the study by Lass et al. [14], used in the pediatric PBPK model validation. The pediatric PBPK model was evaluated against available clinical PK data [14], with predicted plasma concentration profiles for SP, CAN, and TMS accurately capturing observed data following the oral administration of 1 mg/kg of SP suspension in neonates and infants up to 2 years of age (Figure 5). The PBPK model was then applied to simulate SP dosing in pediatrics that would match the unbound systemic exposure to the active compounds in adults following a starting dose of 50 mg (Figure 6A) or a maintenance dose of 100 mg (Figure 6B). Assuming a similar dose–response relationship in adults and pediatrics, our simulations indicated that a dose of 1 mg/kg of SP in infants and neonates matches the total unbound systemic exposure observed after a 50 mg starting dose in adults for treating edema. The simulations also indicate that an SP dose of 2.5 mg/kg for older infants (6 months to 2 years), 2 mg/kg of SP in young infants (1–6 months), and 2.5 mg/kg in neonates (birth to 1 month) are needed to achieve the total unbound systemic exposure equivalent to the standard recommended daily maintenance dose of 100 mg in adults for treating edema.

## 3. Discussion

To date, no dedicated published studies have assessed the efficacy and safety data of SP in pediatrics. Consequently, dosing recommendations for SP are derived solely from clinical experience and case studies ranging from initial doses from 1 to 3 mg/kg/day divided every 6–24 h, up to a maximum of 9 mg/kg/day. Given the limited PK data available in pediatrics, our study aimed to develop an adult PBPK model as a foundation for extrapolation to pediatric populations. This approach accounted for age-related physiological changes and the ontogeny of drug-metabolizing enzymes. Our PBPK model, developed using a middle-out approach, accurately predicted plasma concentration–time profiles and key PK parameters, such as C_max_ and AUC, in adults. Assuming the concentration–response relationship for SP in pediatrics mirrors that of adults, we further extrapolated the model to pediatric patients. By matching systemic exposure in pediatrics to that observed in adults, we proposed loading and maintenance doses for term neonates (up to 1 month), infants aged 1–6 months, and infants aged 6 months to 2 years.

SP is classified as a neutral BCS Class II drug with poor aqueous solubility and high permeability, resulting in low and variable oral bioavailability. Notably, the food effect nearly doubles its exposure [8]. Our PBPK model successfully accounted for the impact of food, enhancing the predictability and clinical relevance of dosing recommendations to improve patient outcomes. Our approach considered that not only SP but also TMS and CAN are active compounds. Early PK studies in adults primarily focused on the PK profile of CAN, under the mistaken assumption that it was the sole active metabolite. This misconception arose from the limitations of fluorometric assays, which lacked specificity and could not distinguish between metabolites. With the advent of HPLC assays, TMS was identified as the main active metabolite of SP [7]. Therefore, the PK data used to verify our model incorporated bioanalytical methods that avoided the specificity limitations of earlier fluorometric assays.

Carboxylesterase is the primary enzyme responsible for converting SP into its metabolites [33]. For modeling purposes, CES1 was assumed to be the primary enzyme involved, given its preferential hydrolysis of carboxylic esters with a larger acyl moiety and its predominant expression in the liver. In contrast, CES2, which primarily hydrolyzes favors smaller acyl groups, is mainly found in the human small intestine [33,34]. To facilitate modeling, SP was assumed to be entirely and directly converted to its active metabolites, bypassing the intermediate 7α-thiospirolactone. While this assumption may affect the time to reach C_max_, the PBPK models provided a consistent representation of the three active compounds demonstrating good predictive performance for single and multiple SP doses. This highlights the utility of the developed models and strengthens our hypothesis regarding SP metabolism. We recognize that bottom-up PBPK modeling involving non-CYP enzymes remains challenging. With proteomics approaches [35], PBPK modeling for non-CYP enzymes has advanced significantly. However, parameter optimization and middle-out approaches have been predominantly applied in these cases [36] due to the uncertainty in population expression levels for some non-CYP enzymes [37].

By incorporating unique patient physiology and drug characteristics, PBPK modeling becomes a powerful tool for predicting drug PK and optimizing pediatric dosing. Clinical trials in infants and neonates face challenges, such as obtaining parental permission and the need to adapt research procedures [38]. By successfully scaling the adult PBPK model to pediatrics, incorporating enzyme ontogeny, and addressing physiological differences, we demonstrated the potential of PBPK modeling to predict PK in pediatrics. However, CES1 activity is lower in children than in adults and maturates gradually [39,40,41], creating limitations for predicting PK in preterm neonates due to insufficient data on CES1 ontogeny during fetal development.

CYP3A7, the primary fetal form of CYP3A, was included in the pediatric model because of its significant expression in neonates, which decreases with age and becomes negligible in adults [33]. The inclusion of CYP3A7 plays a significant role in metabolizing CYP3A4 substrates in neonates. A study by Williams et al. provides detailed information on the relative metabolic capabilities of CYP3A4 and CYP3A7 by comparing Km and Vmax values for a structurally diverse set of molecules (n = 15). On average, the Km values for CYP3A7 were 5.1 times higher than those of CYP3A4, while the Vmax values were 75% lower. This significant difference in metabolic capabilities underscores the need for including CYP3A7 in the pediatric model to reflect the metabolic processes in this age group [42].

Different dosing strategies for SP are proposed for each pediatric subpopulation to achieve a total systemic exposure comparable to the starting daily dose of 50 mg used in adults for treating edema. In the absence of receptor binding data, the proposed dosing strategy considers the relative potencies of the SP metabolites, CAN, and TMS relative to the unchanged compound. This was previously assessed using the urinary log_10_ Na/K biomarker [43]. Although 7α-thio-spironolactone has been identified as another active SP metabolite with a relative potency of 0.26, there are currently no PK data available to characterize its formation in adults and pediatrics. Therefore, the current approach takes into account the exposure to SP, CAN, and TMS—the latter being the major plasma metabolite in humans [43]—and their relative potencies to propose optimal dosing in neonates and infants. Our simulations indicate that doses of 2.5 mg/kg in older infants and neonates and 2 mg/kg in young infants may be necessary to achieve the total unbound systemic exposure equivalent to the standard recommended daily maintenance dose of 100 mg in adults treating edema. The higher recommended doses in neonates compared to younger infants can be attributed to the increased expression of CYP3A7 in neonates. This aligns with the drug labeling recommendations [44], which suggest doses of 1–3 mg/kg for edema and doses up to 9 mg/kg in infants and 7 mg/kg in neonates for resistant ascites conditions. Although the proposed doses assumed a similar PK/PD relationship in adults and pediatrics, neonates are known to present extremely low renal mineralocorticoid receptors at birth [45]. Therefore, SP should be used with caution in neonates, considering the partial resistance to the drug due to the maturation of mineralocorticoid activity.

In summary, our model provides valuable insights for spironolactone dosing in infants up to 2 years old and can be used to further design and optimize clinical studies in pediatrics. However, conducting prospective studies focusing primarily on spironolactone efficacy and safety in infants is warranted to confirm our assumptions and verify our model predictions. It is essential to note some limitations of the current study. First, the developed model is not applicable to preterm neonates due to the unavailability of ontogeny data for CES1 and PON3 during fetal life. Additionally, the preterm population in the simulator lacks the mechanistic absorption model (ADAM), which was used for other age groups (Figure S3, Supplementary Materials). Therefore, extrapolating the current model to pediatrics would require fitting the absorption to a first-order process and collecting data on the maturation of CES1 and PON3 enzymes during fetal development or in preterm neonates. Secondly, some metabolic pathways were lumped for modeling purposes due to the absence of enzyme kinetics data. Lastly, the performance of the pediatric model was only validated with single doses, as no multiple-dose data are available.

## 4. Methods

### 4.1. Software and Modeling Strategy

The PBPK models were developed with the Simcyp™ Simulator (V23). Clinical data from the scientific literature were digitized with WebPlotDigitizer (version 5, https://automeris.io/, accessed on 7 February 2025). Our strategy was to gain knowledge on SP PK by initially building a PBPK model for adults, which would then be extrapolated to a pediatric PBPK model. The adult PBPK model was built and then evaluated against a set of observed plasma profiles and PK parameters not used during model development to validate its parametrization. The model was extrapolated to pediatric populations and verified against available clinical data [14]. Subsequently, the validated pediatric model was used for the prospective prediction of the PK of SP and its active metabolites in term neonates.

The parent-metabolite PBPK model was developed using a middle-out approach [46] employing the Advanced Dissolution, Absorption, and Metabolism (ADAM) model [47] along with the full PBPK distribution model, were employed, following the workflow presented in Figure 1. The SIVA Toolkit (Version 4, Release 1; Certara UK Limited, Sheffield, UK) was used to model available in vitro biorelevant solubility data to predict the potential effect of food on absorption. Clinical PK data [17] were utilized to optimize the disposition parameters reflecting the metabolism of SP into its active metabolites, CAN and TMS.

### 4.2. Model Development for Spironolactone and Its Metabolites in Adults

An extensive literature search was conducted to build the adult parent-metabolite PBPK model of SP and its main active metabolites, CAN and TMS. This search aimed to gather information on physicochemical properties, ADME processes, and clinical studies with PK profiles for the three modeled compounds. Only clinical PK data measured using liquid chromatography (LC) methods were utilized to develop and evaluate the PBPK model. The collected knowledge informed the drug-specific input parameters and the implementation of parameters relevant to ADME processes. Protein binding was implemented using the Simcyp QSARs for the three modeled compounds.

The solubility of SP is pH-independent [48]. The partition coefficient of SP between water and bile salt micelles (K_m:w, unionized_) was estimated from its solubility in biorelevant media FaSSIF and FeSSIF. Tablets were the primary dosage form in all collected PK studies. Consequently, the Diffusion Layer Model (DLM) was employed to describe the dissolution of the active pharmaceutical ingredients (API) particles using the immediate release (IR) solid formulation with a default particle size of 1 µm, implemented as monodispersed particles. All other DLM settings were kept at their default inputs. Because food intake nearly doubled SP’s AUC [8], the Mechanistic Permeability (MechPeff) model [49] was selected since it accounts for regional solubility in fasting and fed state to inform the potential food effect on drug absorption. The intrinsic membrane permeability (P_trans,0_) was initially informed from in vitro Caco2 permeability assays and then used to predict SP’s regional effective gut wall permeability (P_eff,man_). The fraction unbound in the enterocytes (fu_gut_) was set as the default value of 1, based on the reported poor bioavailability of SP [50,51].

The distribution process was modeled using a full PBPK model. The volume of distribution at steady state (Vss) and tissue–plasma partition coefficients (Kp) were predicted based on the Poulin and Theil model (Method 1), which is suitable for neutral compounds like SP. Due to SP’s complex metabolic pathways, it was assumed that SP is directly and entirely converted to its main active metabolites, CAN and TMS, by the carboxylesterase1 (CES1) enzyme. The intrinsic clearance (Cl_int_) of CES1-mediated metabolism of SP was estimated and used as the input for the metabolites CAN and TMS by fitting the parent-metabolite model to the observed plasma PK profiles from the Overdiek et al. study [17]. Initial estimates were informed by the Cl_int_ of the liver, representing its role as a whole-organ metabolism system. The Cl_int_ of the whole liver was split into 20% for CAN and 80% for TMS, as previously reported [10]. All optimizations were performed using the Weighted Least Square algorithm and the Nelder-Mead method. The selection of the Overdiek et al. study for model development was guided by its comprehensive information, including dosing regimens, availability of PK for the SP and the active metabolites CAN and TMS, and the description of food consumption.

Compound files for CAN and TMS were created using drug-dependent model input parameters either collected from the literature or predicted using Quantitative Structure-Activity Relationship (QSAR) models in the simulator. The estimated blood-to-plasma ratio (B/P) value assigned to SP was also used for TMS, as no direct determination of that parameter has been found in the literature for this metabolite. Similarly to SP, the distribution process for CAN and TMS was modeled using a full PBPK model. The Vss and Kp for both CAN and TMS were predicted based on Method 1. Additionally, the in vivo clearances (CLoral) for CAN and TMS were optimized based on the observed mean CLoral following a single dose of 200 mg of SP to healthy volunteers [17]. As TMS is primarily metabolized by the CYP3A4 enzyme [11], the reverse translational tool (RTT) was utilized to estimate the intrinsic CL by CYP3A4 from the in vivo CLoral, assuming 100% hepatic CL by CYP3A4. Similarly, RTT was used to compute intrinsic CL by paraoxonase 3 (PON3) from the in vivo CLoral for CAN.

### 4.3. Model Validation for Spironolactone and Its Metabolites in Adults

The parent-metabolite PBPK model developed for adults was evaluated for its ability to predict plasma concentrations following single and multiple SP doses across nine clinical studies in adults. Virtual populations of 100 healthy volunteers (10 virtual trials of 10 subjects each) were generated for each study based on the demographics of the corresponding simulated study. For studies without specified age range or gender distributions, virtual populations consisted of individuals aged 20 to 50 years, with an equal gender distribution (50% female). Unless otherwise specified, the fed state was assumed as the default condition in the study design.

The performance of the adult PBPK model was assessed both graphically and numerically. Observed plasma concentration–time profiles from adult studies were visually compared to the PBPK model-predicted profiles. Additionally, predicted and observed values for the area under the plasma concentration–time curve from the first to the last data point (AUC_last_) and maximum plasma concentration (C_max_) were compared using goodness-of-fit (GoF) plots.

### 4.4. Model Development for Spironolactone and Its Metabolites in Neonates and Infants

The adult PBPK model was scaled to predict the PK of SP, TMS, and CAN in pediatrics. The fraction unbound of SP, CAN, and TMS was adjusted to pediatrics based on the ratio of abundance of binding protein abundance in infants versus adults [32]. The formulation was updated from solid tablet to suspension, and a sensitivity analysis was conducted to optimize the formulation’s particle size. A polydisperse particle size distribution was selected to better capture the variability in pediatric PK profiles. As SP is a Biopharmaceutics Classification System (BCS) Class II compound, age-related changes in oral drug performance were considered, given its solubility-limited absorption. Therefore, SP solubility was adjusted to account for the milk effect [52]. All other drug-dependent parameters were kept consistent with the adult PBPK model. The pediatric virtual population was then selected in the simulator, with anatomical and physiological parameters adjusted to reflect age-related changes from birth (minimum age: 0 years) to young adults (maximum age: 25 years). The simulator incorporated ontogeny functions for CES1 and CYP3A4 (profile 1) to scale enzyme concentrations in relevant organs. Since CYP3A7 is the predominant fetal form of CYP3A [42], it was included in the model to improve PK predictions for CYP3A substrates in pediatrics. The Cl_int_ of TMS metabolism via CYP3A7 was estimated using term neonates’ data [14], as CYP3A7 expression is highest in neonates and negligible in adults [42]. Due to limited developmental data on the PON3, which catalyzes the hydrolysis of CAN to canrenoic acid, no ontogeny functions were applied for this enzyme. Instead, CL was scaled using age-related effects on physiological parameters. The extrapolated pediatric PBPK model was subsequently used to predict the plasma concentration–time profiles in term infants under 2 years of age following a single oral dose of 1 mg/kg.

### 4.5. Simulations for Optimal Dosing in Pediatrics

The pediatric PBPK model was used to perform simulations. It was assumed that the pharmacokinetic/pharmacodynamic (PK/PD) relationship in pediatrics is similar to that in adults. Simulations were conducted to match the systemic exposure observed in adults. Two simulated adult regimens included a starting oral dose of 50 mg and a recommended maintenance oral dose of 100 mg for treating edema [2,44]. The simulated clinical study encompassed adult subjects aged 18–55 years who received starting oral doses of 50 mg of SP tablets, followed by a maintenance dose of 100 mg daily. For the pediatric population, simulations were carried out in the following age groups: term neonates (0–1 month), young infants (1 month–6 months), and older infants (6 months–2 years). The simulations for SP dosing in infants and neonates ranged from 1 to 3 mg/kg in 0.5 mg increments, based on the recommended dosage range for children [44]. The fraction unbound of SP, CAN, and TMS was calculated for infants and neonates using the method described by McNamara and Alcorn [32]. All other settings for the pediatric PBPK model for SP and its metabolites remained unchanged. Each simulation was carried out with 10 trials of 10 subjects each, resulting in a total of 100 individuals. All simulations were conducted over a period of seven days to ensure that a steady state for the three modeled compounds was reached. The total exposure (AUC) to unbound SP, TMS, and CAN was normalized according to the relative potency of each compound for the renal anti-mineralocorticoid activity in humans. The relative potencies for CAN and TMS were set at 0.08 and 0.33, respectively, compared to SP, based on urinary sodium/potassium concentrations [43]. Simulated AUC unbound on the seventh day for SP, CAN, and TMS in pediatrics were computed for each age group, normalized based on relative potencies, and compared to the PK parameters in adults.

### 4.6. Data Visualization

To effectively manage, analyze, and visualize our data, we employed R^®^ version 4.4.1 [53]. Non-compartmental analysis was performed with the R package NonCompart, version 0.6.0.

## 5. Conclusions

In summary, the PBPK models developed for SP and its major metabolites, CAN and TMS, effectively predicted the observed PK of these compounds in both adults and pediatric patients. The adult PBPK model is a pivotal step toward extrapolating findings to pediatrics, enabling a priori prediction of PK in young infants and term neonates. By defining age-based physiological changes, the effects of breast milk and formula on drug solubility, and the ontogeny of metabolizing enzymes, PBPK models were rationally scaled for pediatrics use. These models provide valuable insights for informing dosing decisions and optimizing therapeutic outcomes in populations where clinical studies pose challenges.

## Figures and Tables

**Table 1:** Clinical PK data was used to develop and validate the PBPK model for spironolactone and its major metabolites in adults.

	Reference	PK Profile	Dosage Regimen	Number, Gender	Age {Years} Mean [Median] (Range)	BW {kg} Mean [Median] (Range)	Food Consumption
Healthy adult PBPK model development	Overdiek et al. [17]	SP CAN TMS	200 mg, SD	4 M	34	76	Fed state
Healthy adult PBPK model validation	Gardiner et al. [10]	SP CAN TMS	100 mg, SD and MD	12 M	[22] (18–39)	[70] (58–89)	Fed state
	Jankowski et al. [18]	SP CAN TMS	100 mg, SD	1 NA	NA	NA	NA
	Overdiek and Markus [8]	SP	200 mg, SD	7 M/2 F	(18–45)	(58–80)	Fasting and fed state
	Vlase et al. [19]	SP CAN	100 mg, SD	24 NA	NA	NA	NA
	Dong et al. * [20]	SP CAN	100 mg, SD	2 NA	NA	NA	Fasting
	Abshagen et al. [21]	CAN	100, SD and MD 500 mg, SD	10 M/10 F	27.2	66.35	NA
	Merkus et al. [22]	CAN	100 mg, SD	6 M/2 F	26	71	NA
	Kojima et al. [23]	CAN	100 mg, SD	10 M	(33–45)	(45–70)	NA
	Lee et al. [24]	SP	50 mg, SD	50 M	NA	NA	NA

SP: spironolactone; CAN: canrenone; TMS: 7α-thiomethylspironolactone; BW: body weight; M: male; F: female; SD: single dose; MD; multiple dose; NA: not provided in the study; * Healthy Chinese population.

**Table 3:** Infant demographics from the study by Lass et al. [14] used in the pediatric PBPK model.

	Infant (1 mon–2 yr)	Infant Subgroup1 (1 mon–6 mon)	Infant Subgroup2 (6 mon–2 yr)	Neonate (Birth–1 mon)	Preterm Neonate
Number of individuals	15	5	10	6	2
Postnatal age	8.5 (1.2–21.4) months	2 (1.2–6) months	12.5 (7.2–21.4) months	9 (3–25) days	8.5 (3–14) days
Gestational age (weeks)	40 (37–41)	39 (37–41)	40 ^#^	40 (38–41)	35
Current weight (kg)	7.8 (2.2–12.6)	4.3 (2.2–5.8)	8.4 (6.4–12.6)	3.4 (3.1–3.7)	2.7 (2.6–2.8)
Gender (M/F)	6/9	1/4	5/5	1/5	1/1
Spironolactone dose administered via (oral route/gastric tube)	11/4	2/3	9/1	2/4	2/0

Data expressed as median and range. ^#^ missing data.

## Data Availability

Input data used to build the model are available in the Supplementary Materials. Model files will be made available by authors on request.

## Supplementary Materials

The following supporting information can be downloaded at: https://www.mdpi.com/article/10.3390/ph18030355/s1, Table S1. Absorption parameters for spironolactone PBPK model; Table S2. Observed and predicted PK parameters in adult healthy volunteers; Figure S1. Model total spironolactone experimental vs. predicted aqueous solubility; Figure S2. PBPK Model verification in adults. Predicted versus observed plasma concentrations over time following oral administration of spironolactone tablets for spironolactone (red), canrenone (blue) and TMS (green). Observed data are shown as circles representing the mean. The solid line represents the population median and the shaded area is the 90% population prediction interval; Figure S3. PBPK Model application in preterm neonates. Reference [54] is cited in the Supplementary Materials.
