# Supplementary material for: Model-Informed Dose Optimization of Spironolactone in Neonates and Infants
Source: Pharmaceuticals (Basel). 2025 Mar 1;18(3):355. doi: 10.3390/ph18030355 (PMC11944562; doi:10.3390/ph18030355)
Supplement: Supplementary file 1 [file pharmaceuticals-18-00355-s001.zip › pharmaceuticals-3490744-supplementary.pdf]

Supplementary material

Model-informed dose optimization of spironolactone in neonates and infants

Authors: Amira Soliman (1,2), Leandro F. Pippa (1), Jana Lass (3,4), Stephanie Leroux (5), Valvanera Vozmediano (1,6), and Natalia De Moraes (1)

Affiliations:

(1) Department of Pharmaceutics, Center for Pharmacometrics and Systems Pharmacology, University of Florida, Orlando, Florida 32827, USA.

(2) Department of Pharmacy Practice, Faculty of Pharmacy, Helwan University, Helwan 11795, Egypt.

(3) Institute of Pharmacy, University of Tartu, Tartu, Estonia.

(4) Pharmacy Department, Tartu University Hospital, Tartu, Estonia.

(5) Univ Rennes, CHU Rennes, Inserm, EHESP, Irset - UMR_S 1085, F-35000 Rennes, France

(6) Model Informed Development, CTI Laboratories, Covington, KY 41011, USA.

List of Tables and Figures

Table S1. Observed and predicted PK parameters in adult healthy volunteers.

Table S2. Observed and predicted PK parameters in infants and neonates.

Figure S1. Model total spironolactone experimental vs. predicted aqueous solubility.

Figure S2. PBPK Model verification in adults. Predicted versus observed plasma concentrations over time following oral administration of spironolactone tablets for spironolactone (red), canrenone (blue) and TMS (green). Observed data are shown as circles representing the mean. The solid line represents the population median and the shaded area is the 90% population prediction interval.

Figure S3. PBPK Model application in preterm neonates.

Table S1: Observed and predicted PK parameters in adults healthy volunteers

Drug	Parameter	Model	Study	Regimen	Observed	Predicted	Pred/Obs ratio
Spironolactone (SP)	AUC (ng.hr/ml)	development	Overdiek et al.[1],	200 mg, SD, Fed	473	481.57	1.018118393
		Validation	Gardiner et al. [2]	100mg, SD, Fed	177	244.15	1.379378531
		Validation	Gardiner et al. [2]	100mg, MD 15days, Fed	231	230.77	0.999004329
		Validation	Jankowski et al. [3]	100mg, SD, Fed	177	259.43	1.465706215
		Validation	Overdiek and Markus. [4]	200mg, SD, Fasting	288	164.76	0.572083333
		Validation	Overdiek and Markus. [4]	200mg, SD, Fed	493	479.89	0.973407708
		Validation	Vlase et al. [5]	100mg, SD, Fed	177	298.14	1.68440678
		Validation	Dong et al* [6]	100mg, SD, Fasting	120	190	1.583333333
		Validation	Lee et al.[7]	50mg, SD, Fed	105.29	125.94	1.196124988
	Cmax (ng/ml)	development	Overdiek et al.[1]	200 mg, SD, Fed	185	143	0.772972973
		Validation	Gardiner et al. [2]	100mg, SD, Fed	72	71.51	0.993194444
		Validation	Gardiner et al. [2]	100mg, MD 15days, Fed	80	72.23	0.902875
		Validation	Jankowski et al. [3]	100mg, SD, Fed	71	78.3	1.102816901
		Validation	Overdiek and Markus. [4]	200mg, SD, Fasting	84	30.85	0.367261905
		Validation	Overdiek and Markus. [4]	200mg, SD, Fed	184	141	0.766304348
		Validation	Vlase et al. [5]	100mg, SD, Fed	37	54.58	1.475135135
		Validation	Dong et al* [6]	100mg, SD, Fasting	41.5	42.53	1.024819277
		Validation	Lee et al.[7]	50mg, SD, Fed	56.36	39.6	0.702625976
Canrenone (CAN)	AUC (ng.hr/ml)	development	Overdiek et al.[1]	200 mg, SD, Fed	3107	2822.39	0.908397168
		Validation	Gardiner et al. [2]	100mg, SD, Fed	1546	1425	0.921733506
		Validation	Gardiner et al. [2]	100mg, MD 15days, Fed	2173	2372.51	1.091813162
		Validation	Jankowski et al. [3]	100mg, SD, Fed	1400	1467.58	1.048271429
		Validation	Abshagen et al. [8]	100mg, SD, Fed	1800	1475.65	0.819805556
		Validation	Abshagen et al. [8]	500mg, SD, Fed	5786	5132	0.886968545
		Validation	Abshagen et al. [8]	100mg, MD for 5days, Fed	2337	2511.76	1.074779632
		Validation	Kojima et al. [9]	100mg, SD, Fed	1403	1435.23	1.022972202
		Validation	Merkus et al. [10]	100mg, SD, Fed	1541	1456.75	0.945327709

Drug	Parameter	Model	Study	Regimen	Observed	Predicted	Pred/Obs ratio
		Validation	Dong et al* [6]	100mg, SD, Fasting	2100	1218.53	0.580252381
		Validation	Vlase et al. [5]	100mg, SD, Fed	2100	1799.36	0.856838095
	Cmax (ng/ml)	development	Overdiek et al.[1]	200 mg, SD, Fed	231	263.5	1.140692641
		Validation	Gardiner et al. [2]	100mg, SD, Fed	155	132.26	0.853290323
		Validation	Gardiner et al. [2]	100mg, MD 15days, Fed	181	172.54	0.953259669
		Validation	Jankowski et al. [3]	100mg, SD, Fed	130	140.98	1.084461538
		Validation	Abshagen et al. [8]	100mg, SD, Fed	133	140.91	1.059473684
		Validation	Abshagen et al. [8]	500mg, SD, Fed	390.4	438.22	1.122489754
		Validation	Abshagen et al. [8]	100mg, MD for 5days, Fed	201.45	198.8	0.986845371
		Validation	Kojima et al. [9]	100mg, SD, Fed	116	134.19	1.156810345
		Validation	Merkus et al. [10]	100mg, SD, Fed	150	137.53	0.916866667
		Validation	Dong et al* [6]	100mg, SD, Fasting	145	132	0.910344828
		Validation	Vlase et al. [5]	100mg, SD, Fed	145	119.24	0.822344828
7α-thiomethyl spironolactone (TMS)	AUC (ng.hr/ml)	development	Overdiek et al.[1]	200 mg, SD, Fed	3880	4154.79	1.070822165
		Validation	Gardiner et al. [2]	100mg, SD, Fed	2242	2112	0.942016057
		Validation	Gardiner et al. [2]	100mg, MD 15days, Fed	2804	2309.14	0.823516405
		Validation	Jankowski et al. [3]	100mg, SD, Fed	1900	2236.42	1.177063158
	Cmax (ng/ml)	development	Overdiek et al.[1]	200 mg, SD, Fed	571	621.18	1.087880911
		Validation	Gardiner et al. [2]	100mg, SD, Fed	359	313.39	0.872952646
		Validation	Gardiner et al. [2]	100mg, MD 15days, Fed	391	312.49	0.799207161
		Validation	Jankowski et al. [3]	100mg, SD, Fed	310	338.4	1.091612903

* healthy Chinese population, Data is expressed as me

Table S2: Observed and predicted PK parameters in infants and neonates

		All infants (1mon-2yr) N=15		Young infants (1mon-6mon) N=5		Old infants (6mon-2 yr) N=10		Neonate (birth-1mon) N=6	
		**AUC (ng.h/ml)**	**Cmax (ng/ml)**	**AUC (ng.h/ml)**	**Cmax (ng/ml)**	**AUC (ng.h/ml)**	**Cmax (ng/ml)**	**AUC (ng.h/ml)**	**Cmax (ng/ml)**
**SP**	**Predicted**	73.40	16.16	97.95	20.71	68.77	14.55	137.00	26.68
	**Observed**	93.11	20.28	93.11	43.2	87.03	18.965	152.445	26.67
	**Ratio**	0.79	0.80	1.05	0.48	0.79	0.77	0.90	1
**CAN**	**Predicted**	420.56	31.09	518.40	39.72	419.47	30.98	347.52	27.02
	**Observed**	447.85	25.15	425.74	25.25	460.34	27.435	332.715	21.365
	**Ratio**	0.94	1.24	1.22	1.57	0.91	1.13	1.04	1.26
**TMS**	**Predicted**	673.67	76.60	876.38	89.12	614.60	73.38	284.98	42.55
	**Observed**	765.815	81.25	1050.46	95.14	528.74	68.735	425.265	37.755
	**Ratio**	0.88	0.94	0.83	0.94	1.16	1.07	0.67	1.13

Data expressed as medians

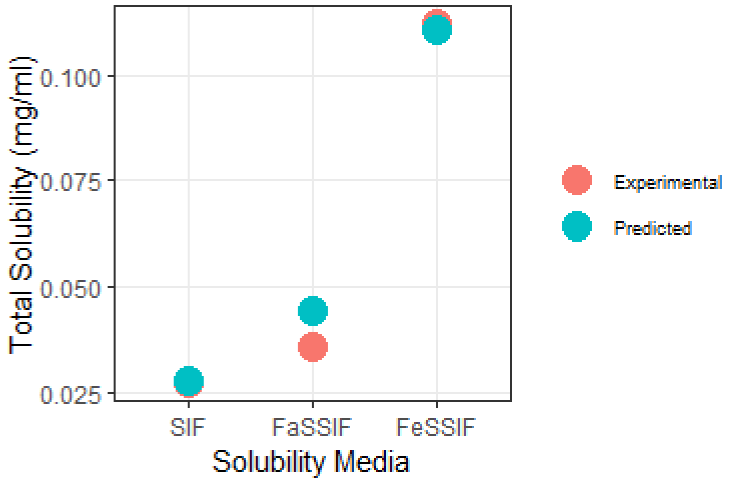

Figure S1. Model total spironolactone experimental vs. predicted aqueous solubility.

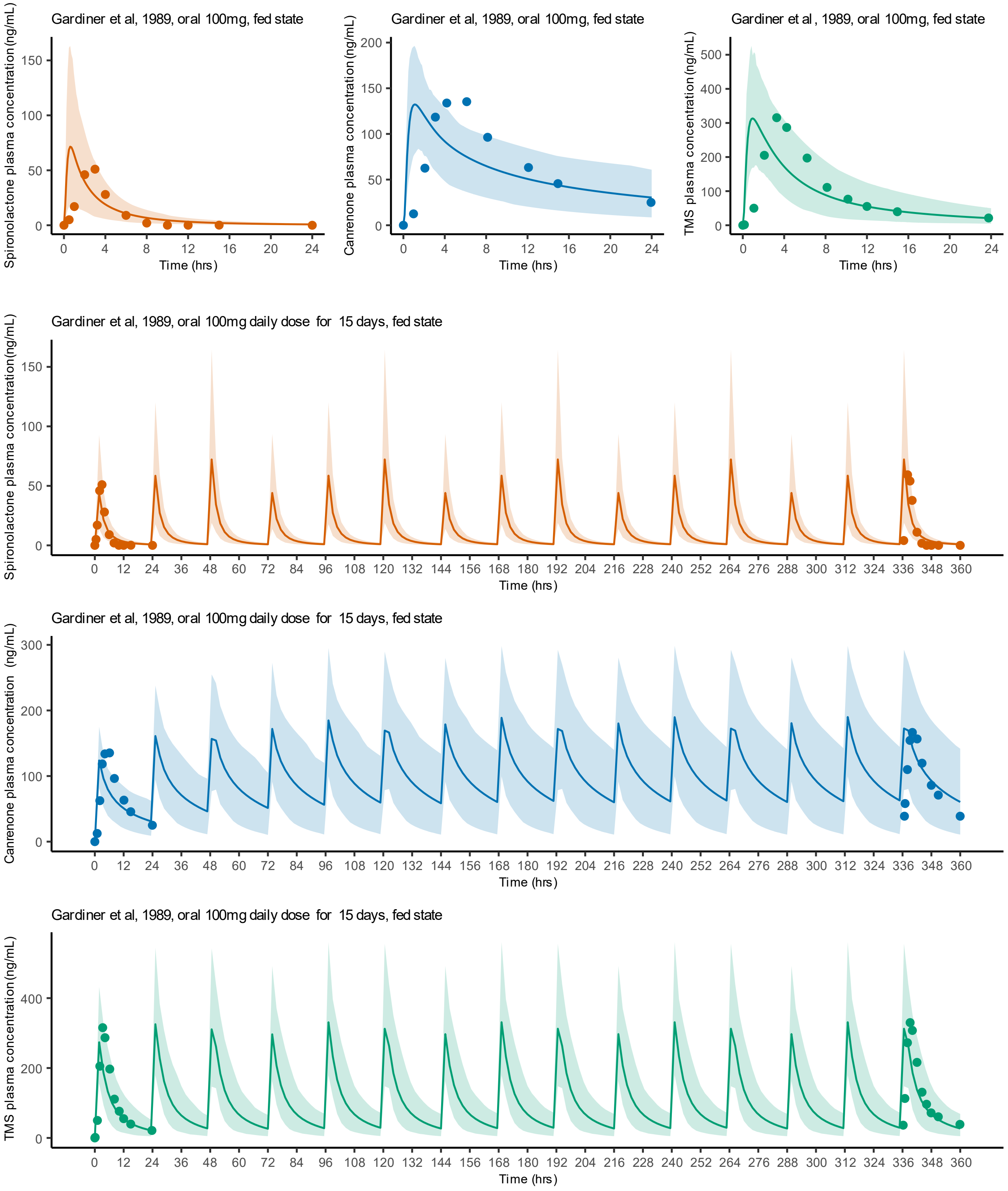

Figure S2. PBPK Model verification in adults. Predicted versus observed plasma concentrations over time obtained after oral administration of spironolactone tablets for spironolactone (red), canrenone (blue) and TMS (green). Observed data are shown as circles representing mean. Solid line represents the population median and the shaded area the 90% population prediction interval.

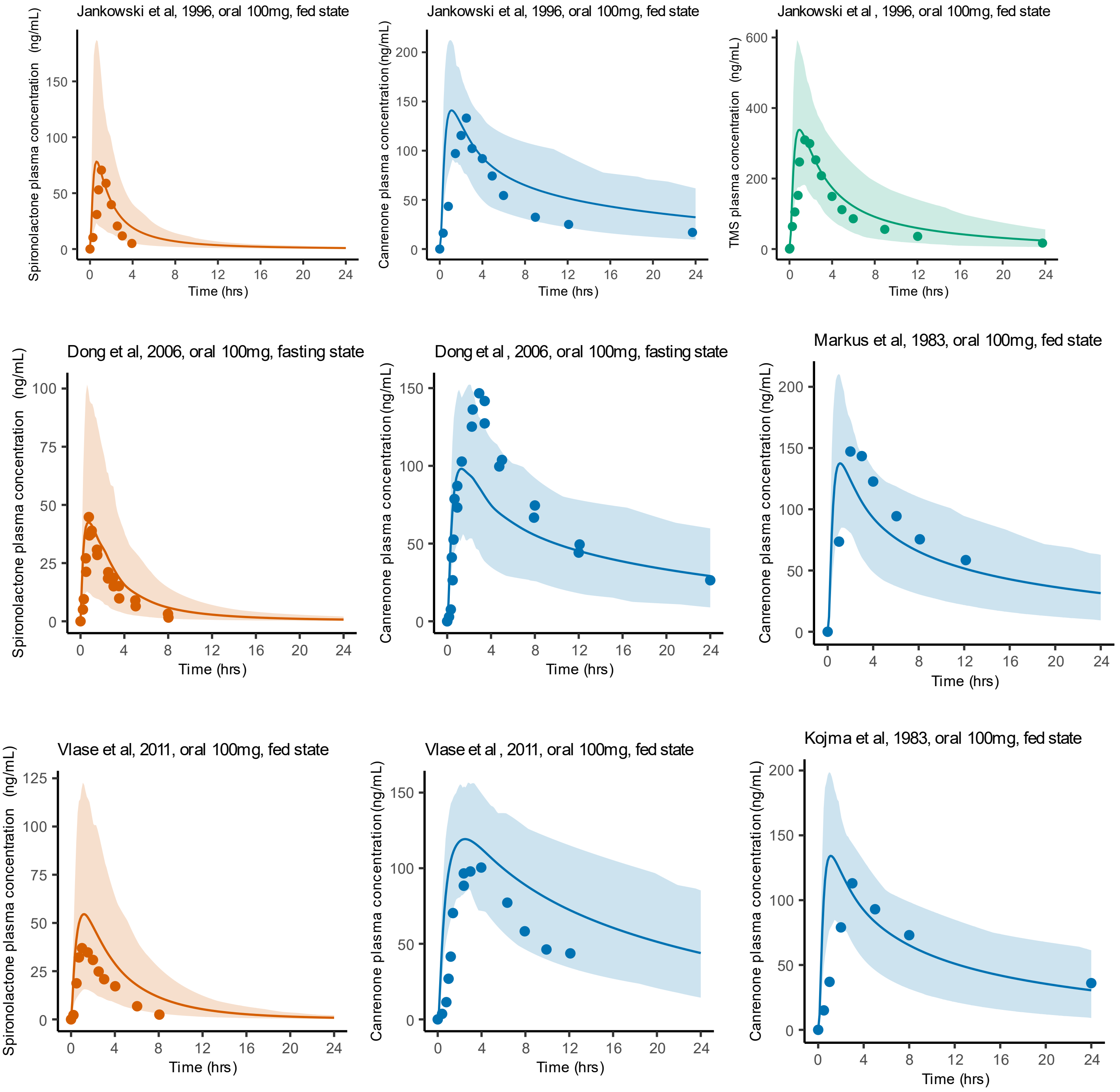

Figure S2. PBPK Model verification in adults. Predicted versus observed plasma concentrations over time following oral administration of spironolactone tablets for spironolactone (red), canrenone (blue) and TMS (green). Observed data are shown as circles representing the mean. The solid line represents the population mean and the shaded area is the 90% population prediction interval.

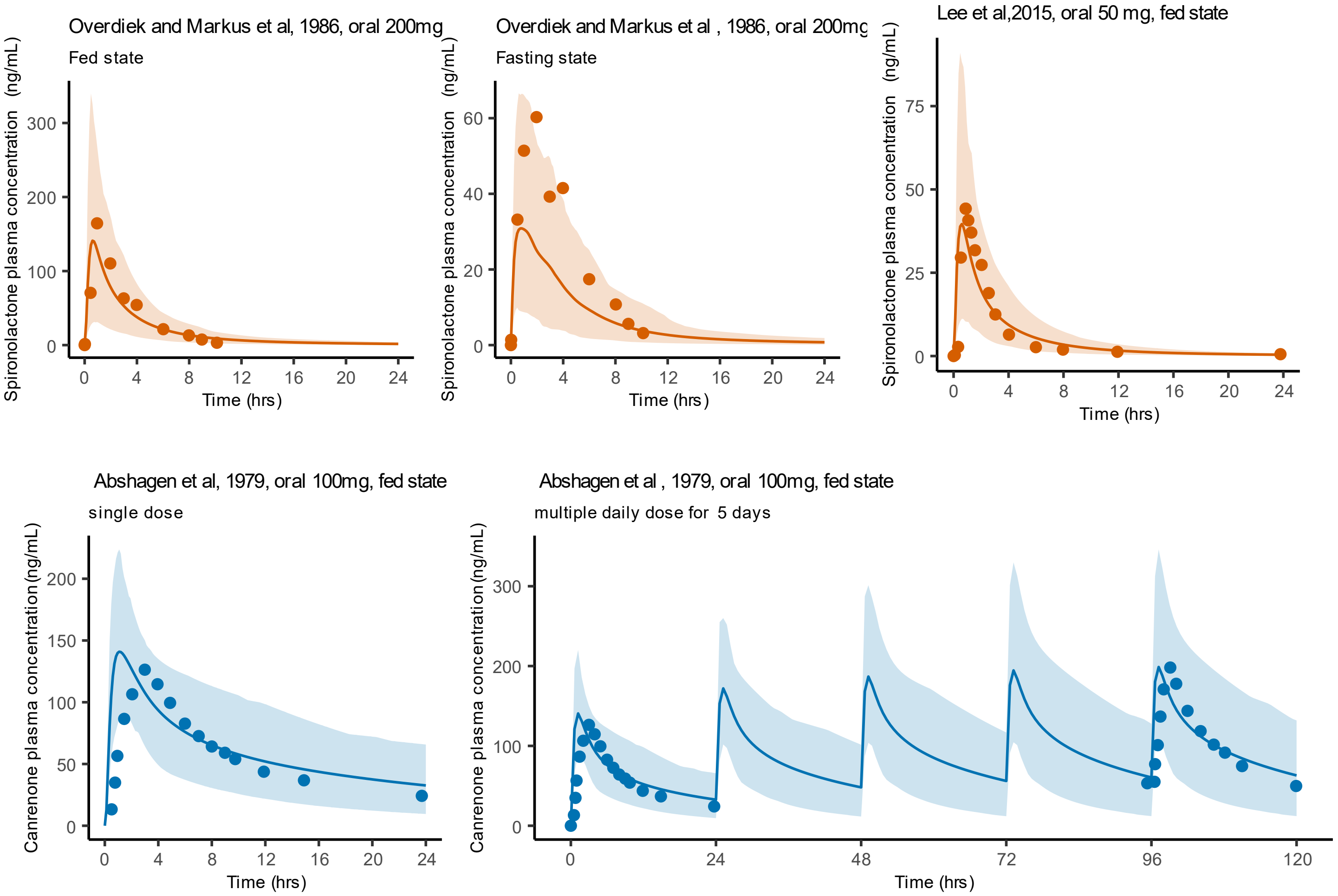

Figure S2. PBPK Model verification in adults. Predicted versus observed plasma concentrations over time obtained after oral administration of spironolactone tablets for spironolactone (red), canrenone (blue) and TMS (green). Observed data are shown as circles representing mean. Solid line represents the population mean and the shaded area the 90% population prediction interval.

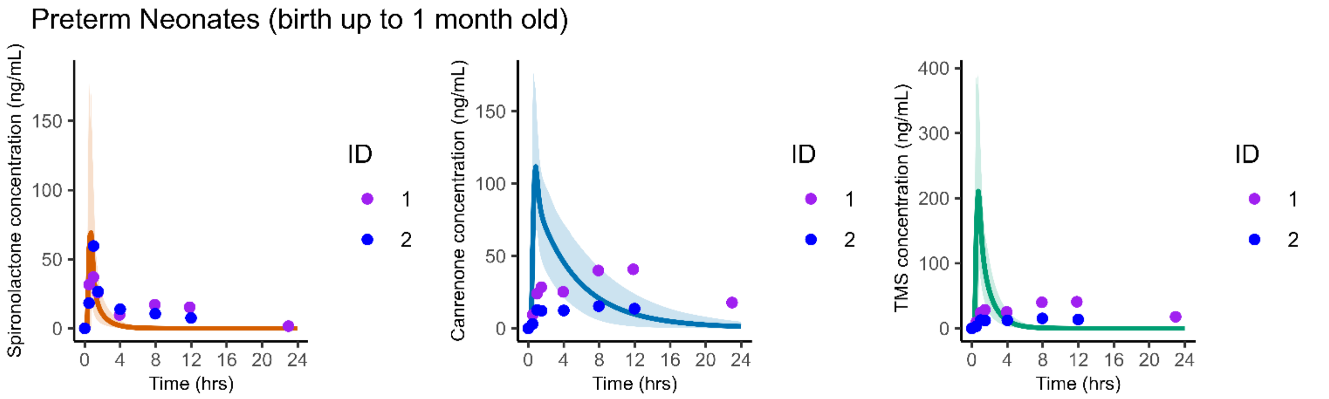

Figure S3. PBPK Model application in preterm neonates. Predicted versus observed plasma concentrations over time obtained after oral administration of 1mg/kg of spironolactone suspension for spironolactone, canrenone and TMS. Observed data; Lass et al. [16] are shown as circles representing mean. Solid line represents the population mean for spironolactone (red), canrenone (blue) and TMS (green) and the shaded area the 90% population prediction interval. First absorption model is applied as the absorption model.

References:

1. Overdiek, H.W.P.M.; Hermens, W.A.J.J.; Merkus, F.W.H.M. New Insights into the Pharmacokinetics of Spironolactone. *Clin. Pharmacol. Ther.* **1985**, *38*, 469–474. https://doi.org/10.1038/clpt.1985.206.
2. Gardiner, P.; Schrode, K.; Quinlan, D.; Martin, B.K.; Boreham, D.R.; Rogers, M.S.; Stubbs, K.; Smith, M.; Karim, A. Spironolactone Metabolism: Steady-State Serum Levels of the Sulfur-Containing Metabolites. *J. Clin. Pharmacol.* **1989**, *29*, 342–347. https://doi.org/10.1002/j.1552-4604.1989.tb03339.x.
3. Jankowski, A.; Skorek-Jankowska, A.; Lamparczyk, H. Simultaneous Determination of Spironolactone and Its Metabolites in Human Plasma. *J. Pharm. Biomed. Anal.* **1996**, *14*, 1359–1365.
4. Overdiek, H.W.P.M.; Merkus, F.W.H.M. Influence of Food on the Bioavailability of Spironolactone. *Clin. Pharmacol. Ther.* **1986**, *40*, 531–536. https://doi.org/10.1038/clpt.1986.219.
5. Vlase, L.; Imre, S.; Muntean, D.; Achim, M.; Muntean, D.L. Determination of Spironolactone and Canrenone in Human Plasma by High-Performance Liquid Chromatography with Mass Spectrometry Detection. *Croat. Chem. Acta* **2011**, *84*, 361–366. https://doi.org/10.5562/cca1761.
6. Dong, H.; Xu, F.; Zhang, Z.; Tian, Y.; Chen, Y. Simultaneous Determination of Spironolactone and Its Active Metabolite Canrenone in Human Plasma by HPLC-APCI-MS. *J. Mass Spectrom.* **2006**, *41*, 477–486. https://doi.org/10.1002/jms.1006.
7. Lee, J.H.; An, T.G.; Kim, S.J.; Shim, W.S.; Lee, K.T. Development of Liquid Chromatography Tandem Mass Spectrometry Method for Determination of Spironolactone in Human Plasma: Application to a Bioequivalence Study of Daewon Spiracton Tablet® (Spironolactone 50 Mg). *J. Pharm. Investig.* **2015**, *45*, 601–609. https://doi.org/10.1007/s40005-015-0197-9.
8. Abshagen, U.; Besenfelder, E.; Endele, R.; Koch, K.; Neubert, B. Kinetics of Canrenone after Single and Multiple Doses of Spironolactone. *Eur. J. Clin. Pharmacol.* **1979**, *16*, 255–262. https://doi.org/10.1007/BF00608404.
9. Kojima, K.; Yamamoto, K.; Fujioka, H.; Kaneko, H. Pharmacokinetics of Spironolactone and Potassium Canrenoate in Humans. *J. Pharmacobio-Dyn.* **1985**, *8*, 161–166. https://doi.org/10.1248/bpb1978.8.161.
10. Merkus, F.W.H.M.; Overdiek, J.W.P.M.; Cilissen, J.; Zuidema, J. Pharmacokinetics of Spironolactone After a Single Dose: Evaluation of the True Canrenone Serum Concentrations During 24 Hours. *Clin. Exp. Hypertens. Part A Theory Pract.* **1983**, *5*, 239–248. https://doi.org/10.3109/10641968309048824.
